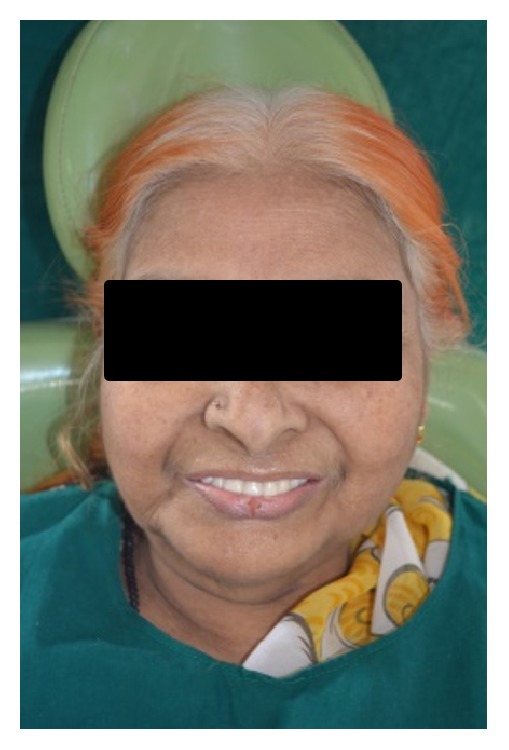# Prosthodontic Management of Xerostomic Patient: A Technical Modification

**DOI:** 10.1155/2016/8905891

**Published:** 2016-02-10

**Authors:** Haraswarupa Gurkar, Omprakash Yadahally Venkatesh, Jagadeesh Mandya Somashekar, Muthuraj Hariharapura Lakshme Gowda, Madhavi Dwivedi, Ishani Ningthoujam

**Affiliations:** Department of Prosthodontics, Farooqia Dental College and Hospital, Mysore 570021, India

## Abstract

Xerostomia is often a contributing factor in both minor and serious health problems. It can affect nutrition and dental as well as psychological health. Common problems faced by such patients are glossitis, mucositis, angular cheilitis, dysgeusia, and difficulty in chewing and swallowing. One of the major problems associated with xerostomic patients is the poor tolerance and retention of removable dental prostheses because of thin dry atrophic mucosa and lack of a saliva film. This paper describes a new technique of incorporating a salivary reservoir in the maxillary complete denture. The salivary reservoir fabricated by this technique provided good lubrication of the oral tissues and was easily cleansed by the wearer and was fabricated from routine denture materials.

## 1. Introduction

Saliva is a viscous, transparent liquid secreted by cells of the salivary glands. It plays a critical role in retention of dentures due to its lubricating function and, thus, dry mucosa often leads to compromise in the retention of prosthesis. Furthermore, salivary flow also facilitates the mastication and food bolus formation and its swallowing. It also aids in speech, mastication, or general oral health and function [1].

Xerostomia, a clinical condition caused by a decrease in the production of saliva, may present itself as a local symptom, as part of a systemic disease such as Sjogren's syndrome, diabetes, and alcoholism, or as a side effect of medications and other conditions such as menopause, following therapeutic radiation to the head and neck regions and vitamin deficiencies [2–6]. A temporary decrease could be from emotional reaction or sialolithiasis [1]. Edentulous patients suffering from xerostomia may complain not only of dry mouth, but also of difficulty in normal functions like eating, speaking, swallowing, and so forth and increased susceptibility to infections. Extreme discomfort in wearing dentures is a common complaint [2, 5].

Sugar-free gum or candies/lozenges may help to increase salivary output, and also drinking water on a regular basis [2–4, 6, 7]. Symptomatic relief can be provided by treatment with parasympathomimetics such as pilocarpine hydrochloride or neostigmine bromide [4, 6, 7]. Artificial saliva and salivary substitutes are other means of managing xerostomia. Artificial saliva acts by humidifying and lubricating the dehydrated oral mucosa. Saliva substitutes mainly consist of aqueous solutions containing the same mineral salts as those found in human saliva. To provide easier application of artificial saliva, an intraoral saliva reservoir in the hollowed lingual flange of a mandibular denture or palatal reservoir is also the technique of choice [6].

## 2. Case Report

**Figure 1:**
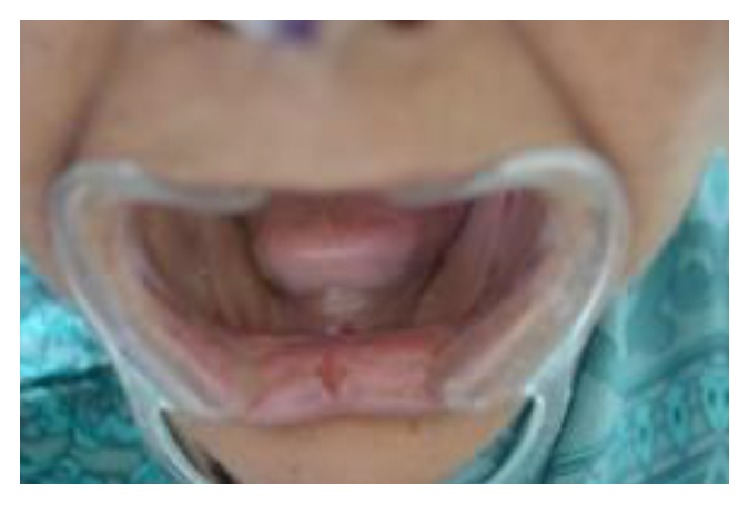


A 65-year-old edentulous female patient reported to Department of Prosthodontics, Farooqia Dental College, having complaint of dryness of mouth and severe discomfort while speaking and eating. Intraoral examination revealed maxillary and mandibular edentulous residual ridges, severely resorbed mandibular ridges, areas of irritation associated with the maxillary denture, dry tongue, and minimal frothy saliva in the floor of the mouth (Figure 1). Patient's mouth was noted to be very dry with cracks at the corner of the mouth and lips. Medical history revealed that she was on medications for hypertension. The patient's general practitioner was also contacted and the medications were reduced or altered to reduce the xerostomia. She had been advised to use salivary substitute regularly and frequently drink water to overcome the dryness and discomfort.

## 3. Procedure

### 3.1. Clinical Steps (Figures 2–5)

**Figure 2:**
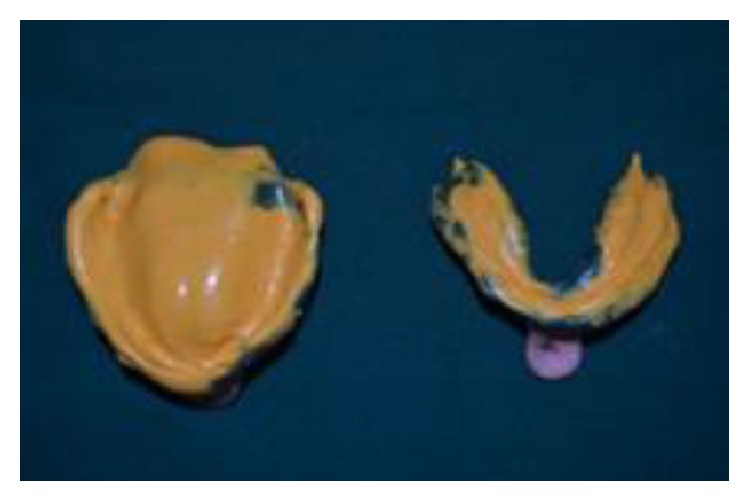


**Figure 3:**
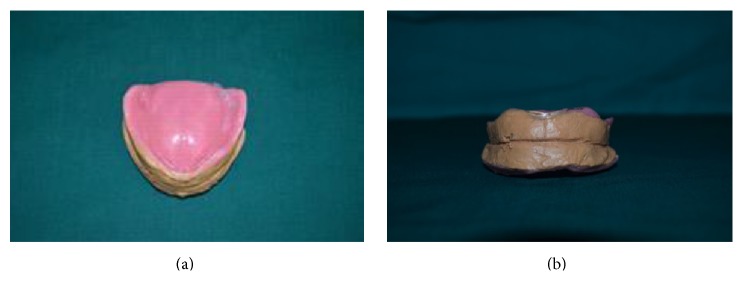


**Figure 4:**
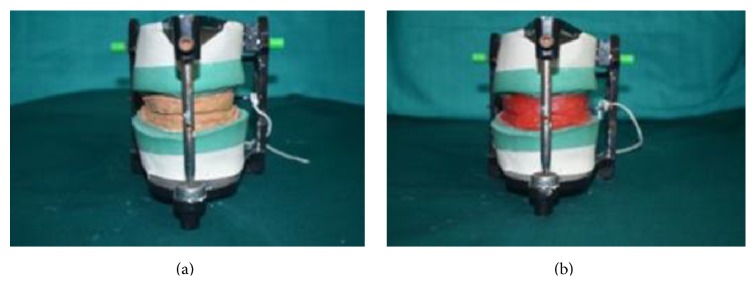


**Figure 5:**
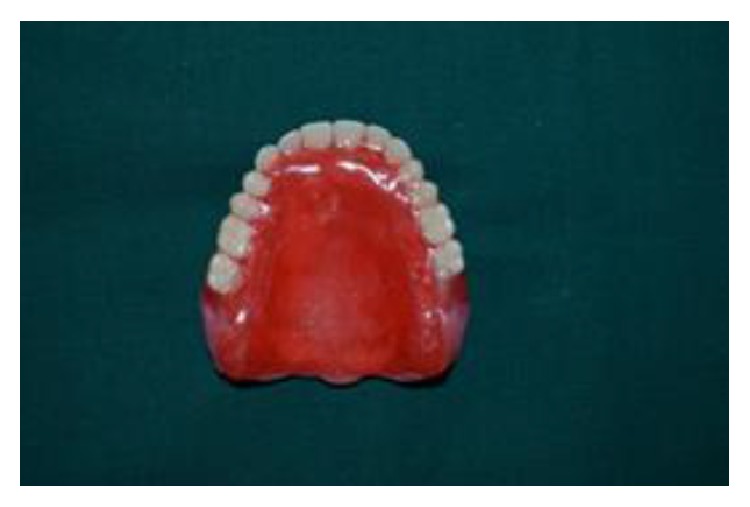


(1) Primary impression was made in irreversible hydrocolloid.

(2) Custom trays were fabricated, border moulding was done, and secondary impressions were made in light body elastomeric impression material (Aquasil Ultra LV Dentsply Caulk) (Figure 2).

(3) Occlusal rims were fabricated using impression compound (DPI Pinnacle) and jaw relation was obtained using closed mouth technique using temporary tissue liner material (Visco-gel Dentsply CE) on the tissue surface of the maxillary and mandibular temporary denture bases for their better stability (Figure 3).

(4) Impression compound occlusion rims were replaced with modeling wax (Hindustan Modelling Wax Number 2, Hyderabad, India) occlusion rims and tissue relining material with cold cure acrylic resin (DPI RR cold cure) (Figure 4).

(5) Teeth were arranged on occlusal rims and palatal surface of the maxillary denture base was covered by the wax to the thickness of 6 mm. Try-in was done using temporary tissue liner material on the polished surface and was allowed to stand in patient's mouth for 30 mins to allow recording the functional movements of tongue and to check for retention, stability, and speech (Figure 5).

### 3.2. Laboratory Procedures (Figures 6–14)

**Figure 6:**
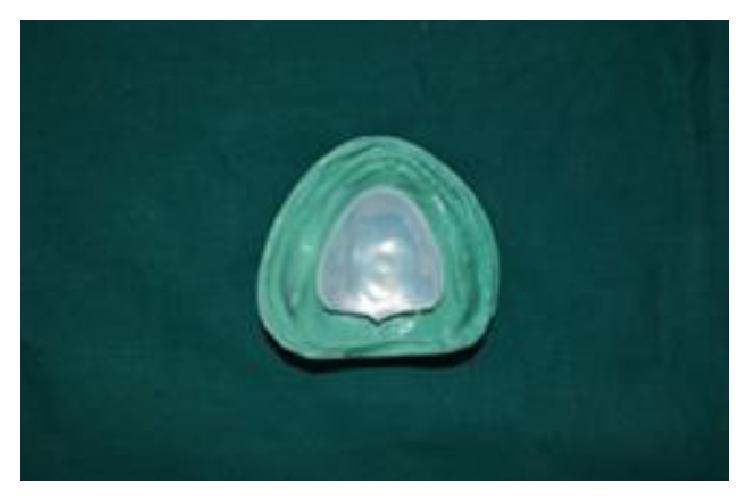


**Figure 7:**
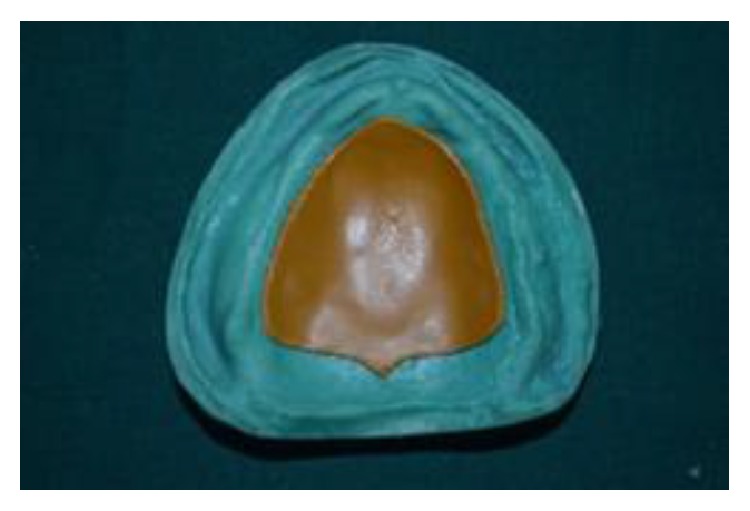


**Figure 8:**
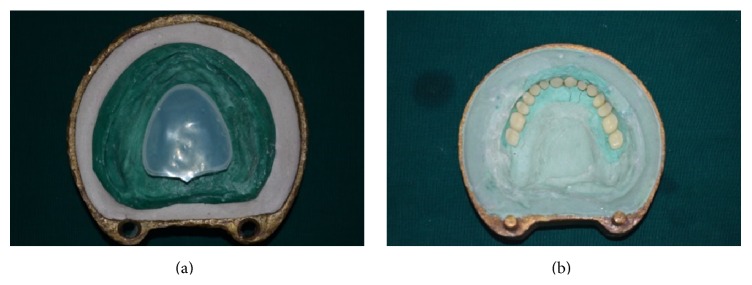


**Figure 9:**
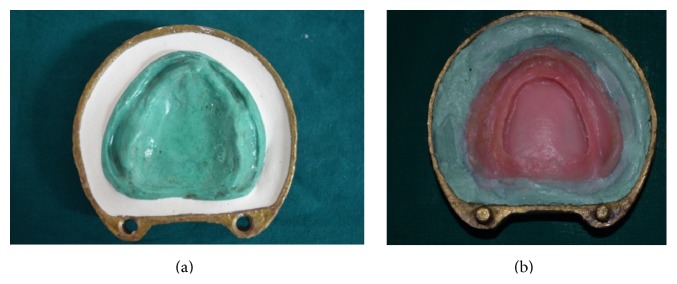


**Figure 10:**
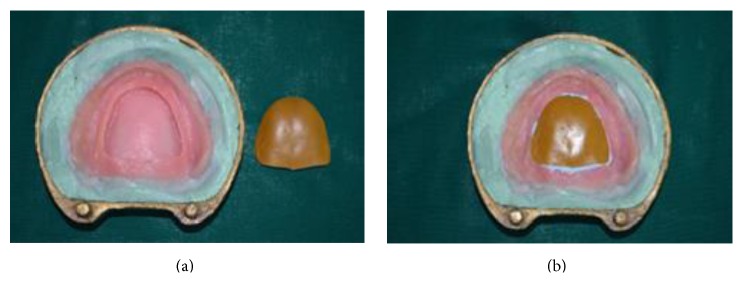


**Figure 11:**
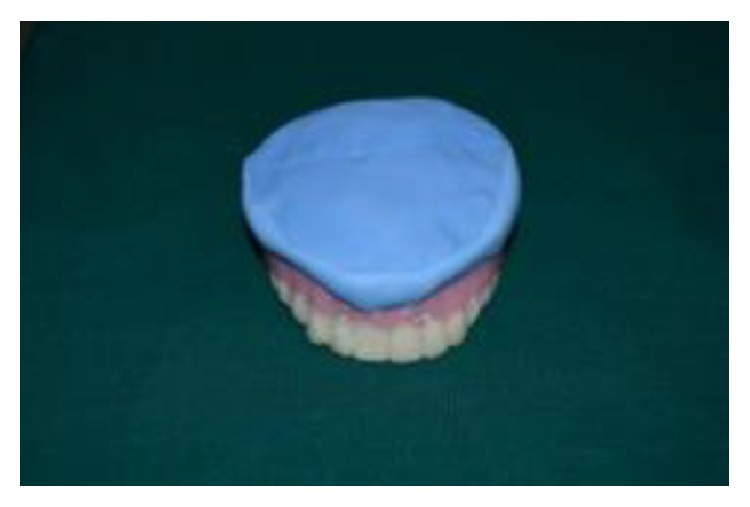


**Figure 12:**
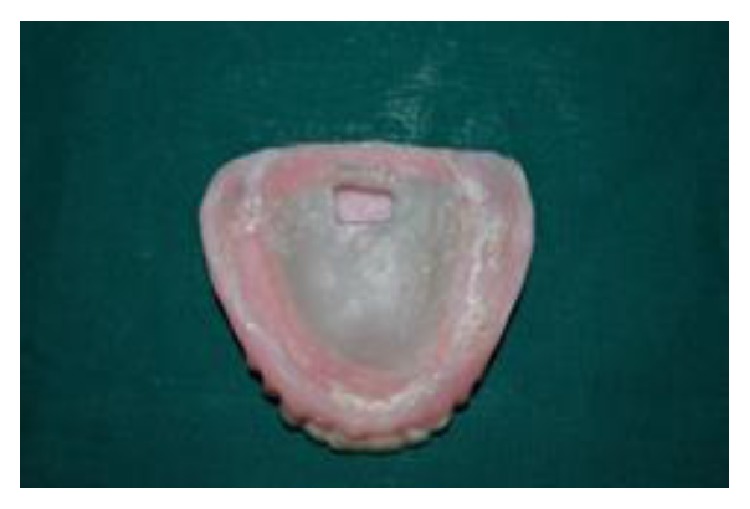


(6) Vacuum formed thermoplastic sheet of 5 mm was heat pressed and cut 2-3 mm short of crest of maxillary ridge on palatal surface and posteriorly just anterior to vibrating line on the maxillary master cast to relieve posterior palatal seal and this sheet spacer was later used at the processing stage (Figure 6).

(7) Shellac base plate (Supernal Base plate, Lucknow, India) of 1 mm thickness was adapted over the maxillary cast and cut into the same size and shape as that of thermoplastic sheet for later use (Figure 7).

(8) Mandibular complete denture was processed in the conventional manner.

(9) For maxillary trial denture which has a thickness of 6 mm (step number (5)), flasking and dewaxing were done in conventional manner. Heat pressed thermoplastic sheet was placed over the cast. Heat cure acrylic resin (Acralyn-“H”, Mumbai, India) was packed into the mould. Heat curing was done in conventional manner (Figure 8).

(10) After heat curing was completed, flask was opened (Figure 9):

- Base part of flask-containing maxillary master cast (Figure 9(a)).
- Counterpart of the flask-containing teeth, polished surface of denture made of pink heat cure acrylic resin and tissue surface of the denture with space created by thermoplastic sheet of 5 mm (Figure 9(b)).

(11) Space of 5 mm created after retrieval of thermoplastic sheet was filled with the elastomeric putty material of thickness 4 mm (GC Flexceed) and closed with the shellac base plate lid of thickness 1 mm on the tissue surface of denture later to create space for clear heat cure acrylic resin (Acralyn-“H”, Mumbai, India) and the base flask was closed with its counterpart (Figure 10).

(12) After the setting of putty material the base flask was opened and any excess putty and also shellac base plate lid (1 mm) were removed. It was again packed with clear heat cure acrylic resin to get the tissue surface of the denture and base flask was closed and it was heat cured again in the conventional manner.

(13) Both the dentures were then finished and polished. Now the processed maxillary denture has

- pink heat cure acrylic on the polished surface (1 mm);
- elastomeric putty material in-between (4 mm);
- clear heat cure acrylic on the tissue surface (1 mm).

(14) Before removing putty spacer, an index impression of the tissue surface of denture was made using putty elastomeric impression material (Figure 11).

(15) The putty spacer was removed by creating a hole on the tissue surface of the denture as shown in Figure 12.

(16) After removal of putty spacer, the hole was closed using cold cure clear acrylic material (DPI-Heat Cure, Mumbai) using the putty index (Figure 13).

(17) Two holes were made: one bigger inlet hole at the tissue surface posteriorly and another smaller outlet hole on the polished surface on anterior palatal on mid-palatine raphae (Figure 14).

(18) Through the inlet hole artificial saliva was injected using 18 mm gauge needle and outlet was in the diameter of 26 mm gauge needle.

(19) The maxillary and mandibular dentures were checked in the patient's mouth for their retention, stability, occlusion, and border extensions and they were finally inserted and the patient was instructed for routine for denture and oral hygiene maintenance and recalled after one week (Figure 15). She was given instructions about how to fill the artificial salivary substitute through the inlet.

## 4. Discussion

Depending on the etiology of the xerostomia, various treatment aspects are available as mentioned above [4]. However 2 or 3 methods are employed to make the prosthesis successful. The goal in management of the xerostomia is to reduce the suffering from the disease and make wearing of dentures and performing normal oral functions comfortable for the patients. At the same time priority should be given to retention and stability of the dentures [4]. In order to enhance retention in xerostomic patients oral moisturizers, denture adhesives, denture reservoirs, soft liners, various denture bases, and various surface treatments are used in order to relieve them from the effects of xerostomia [7–9].

This case report describes the technique of fabrication of the maxillary denture with salivary reservoir. Xerostomic patients can benefit immensely from it as reservoir chamber allows for more controlled flow of artificial saliva [3, 9]. The thickness of the palate was increased primarily in the palatal vault area with minimum increase at the periphery of the palate [4]. Volume of the reservoir was 4.5 mL with its working duration of 2 to 2.5 hours.

Its advantages are easy visibility of the level of salivary substitute in the chamber, easy accessibility to reservoir by the dentist [3], better retention and stability of the dentures, and functional impression of tongue on polished surface which makes room for tongue movements.

Limitations of this technique are that it requires bulky dentures, artificial saliva must be mechanically introduced into the dentures by the patient at regular intervals [5], and it requires additional laboratory steps [3]. The dentures and the reservoir require meticulous cleaning, and patients were instructed in the use of a disposable syringe for flushing with 2% sodium hypochlorite and refilling the chamber [4, 5]. Artificial saliva is contraindicated in conditions like asthma, iritis, and glaucoma as they cause tachycardia, bradycardia, sweating, and increased smooth muscle tone [8].

## 5. Conclusion

Prosthodontics management of xerostomic patient has been a challenging task for the dentist. But this case report provides various methods of treatment that cater to both the need of xerostomia and lack of retention due to resorbed ridges in completely edentulous patients. Since the quality and quantity of saliva have an important role in the success of complete dentures, the patients with hyposalivation need to be treated to prevent adverse effects on the oral mucosa [5, 6].

## Figures and Tables

**Figure 13:**
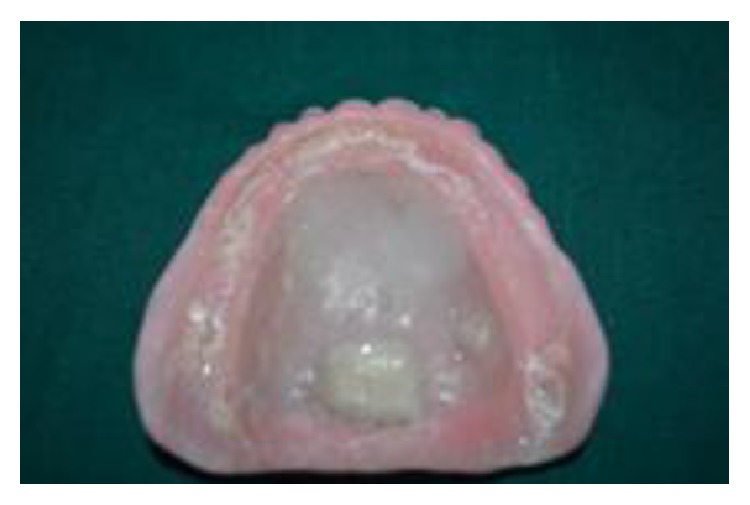


**Figure 14:**
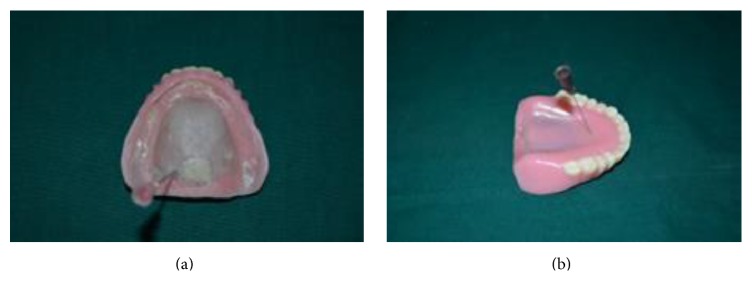


**Figure 15:**